# Supplementary material for: Multitask Approach to Localize Rhizobial Type Three Secretion System Effector Proteins Inside Eukaryotic Cells
Source: Plants (Basel). 2023 May 28;12(11):2133. doi: 10.3390/plants12112133 (PMC10255152; doi:10.3390/plants12112133)
Supplement: Supplementary file 1 [file plants-12-02133-s001.zip › plants-2329835-supplementary.pdf]

Multitask approach to localize rhizobial type three secretion system effector proteins inside eukaryotic cells.

Irene Jiménez-Guerrero, Francisco Javier López-Baena and Carlos Medina*

Departamento de Microbiología, Universidad de Sevilla, Avenida de Reina Mercedes, 6, 41012 Sevilla, Spain

*Correspondence cmedina1@us.es; Tel.: (+34-954554330)

Supplementary Contents:

Supplementary methods.

Supplementary figure S1.

Supplementary Table S1-S2

Supplementary references

Supplementary methods.

Expression and purification of the fusion proteins

The HH103 *nopL* gene was amplified by PCR using the specific primers nopLEX_F and nopLEX_R (Supporting information Table S2). The amplified DNA fragment was cloned into the pGEX-4T-1 vector, adding an *in frame* GST tag to the N-terminus of NopL. Plasmid pMUS1135 was then transformed into *E. coli* BL21(DE3) following the methodology described by [1].

Expression of the NopL protein was performed following the general protocols described by [2]. Briefly, *E. coli* BL21(DE3) carrying plasmid pMUS1135 was inoculated in 5 ml of LB supplemented with ampicillin and grown overnight at 37 ºC. Cultures were transferred to 100 ml of LB containing the same antibiotic and incubated at 37 ºC until the O.D. at 600 nm reached 0.6-0.8. Expression was induced with 1 mM isopropyl β-D-1-thiogalactopyranoside (IPTG) and after 5 h of induction, cells were harvested by centrifugation at 3500 g for 20 min at 4 ºC.

Proteins were purified following the protocol described by [3]. Briefly, cells were lysed by sonication in 3 ml of a buffer containing 20 mM Tris-HCl pH 7.5, 100 mM NaCl, 0.02% Tween 20, 5 mM EDTA, 1 mM EGTA, and the protease inhibitors quimostatin (0.01 µg μl^{-1}), leupeptin (2 µg μl^{-1}) and 1 mM PMSF. After centrifugation at 10000 ***g*** for 15 min at 4 ºC, 1 ml of the supernatant lysate was incubated at 4 ºC for at least 1 h in a 133 µl column of Glutathione sepharose™ 4B resin (GE Healthcare, USA), previously equilibrated. Then, the column was washed at least 3 times with 500 µl of a 20 mM Tris-HCl pH 7.5, 100 mM NaCl, 5 mM EDTA, 1 mM EGTA and 5 mM DTT buffer (centrifugation at 500 g for 5 min at 4 ºC). Finally, the recombinant protein was eluted adding 50 µl of a buffer containing 50 mM Tris-HCl pH 8.0 and 10 mM reduced glutathione (Sigma Aldrich, USA) and centrifuging at 500 *g* for 5 min at 4 ºC (twice). Proteins were further concentrated using Amicon Ultra-4 (10000 MWCO) centrifugal filter tubes (Merck Millipore, USA) following the manufacturer’s instructions. Proteins were stored at -20 ºC adding glycerol at 25% (v/v) and quantified by the method of Bradford [4] employing BSA as standard.

Protein purification was analysed by SDS-PAGE using the discontinuous buffer system of [5]. Electrophoresis was performed on SDS 12% (w/v) polyacrylamide gels and proteins were visualized using PageBlue (Thermo Fisher Scientific, USA) following the manufacturer's instructions.

For immunostaining, proteins were separated and electroblotted to Immuno-Blot PVDF membranes (Bio-Rad, USA) using a Mini Trans-Blot electrophoretic transfer cell (Bio-Rad, USA). Membranes were blocked with TBS containing 2% (w/v) BSA and incubated with antibodies raised against NopL diluted 1:1000 in the same solution. Anti-rabbit immunoglobulin antibody (AP-conjugate) was used as secondary antibody. Reaction results were visualized using NBT-BCIP **(Supplementary Figure S1).**

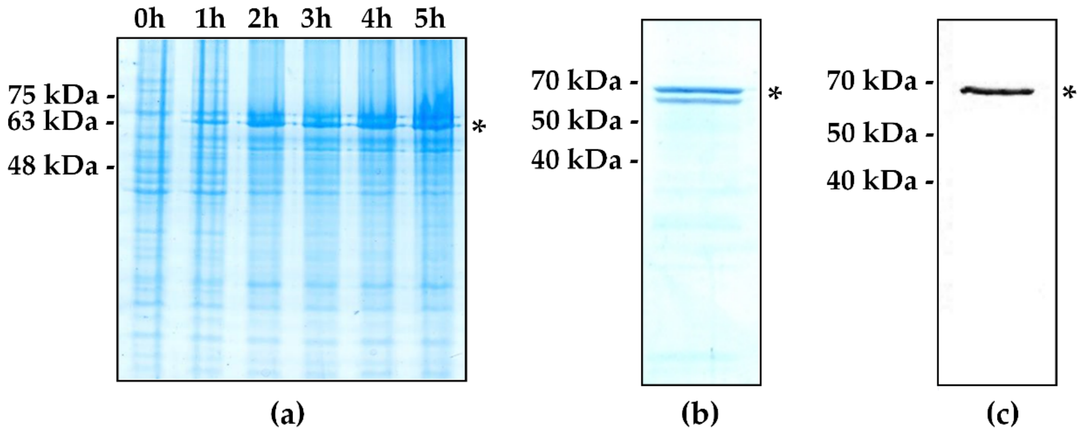

Supplementary Figure S1. ***In vitro*** **expression and purification of recombinant NopL protein in** ***Escherichia coli.*** (a) Expression of NopL from *Sinorhizobium fredii* HH103 in *E. coli* BL21(DE3) at 0, 1, 2, 4 and 5 h after induction by 1 mM IPTG. (b) Purification of NopL 5 h after induction by 1 mM IPTG. (c) Immunodetection of NopL using an anti-NopL primary antibody. Asterisks indicate NopL. Molecular masses (kDa) of the marker are shown on the left in each image.

Supplementary Table S1. Strains and plasmids used in this study.

**Strain/plasmid**	**Relevant properties**	**Source or reference**
Sinorhizobium fredii		
HH103	Parental strain; Rif^R	[8]
Escherichia coli		
DB3.1	*gyrA462*, *endA1*, Δ(*sr1-recA*), *mcrB*, *mrr*, *hsdS20*, *glnV44* (=*supE44*), *ara14*, *galK2*, *lacY1*, *proA2*, *rpsL20*, *xyl5*, *leuB6*, *mtl1*	Invitrogen, USA
DH5α	*supE44*, Δ*lacU169*, *hsdR17*, *recA1*, *endA1*, *gyrA96*, *thi-1*, *relA1*, Nx^R	[2]
BL21(DE3)	*E. coli* B F$^-$*dcmompThsdS* ($r_B^- m_B^-$) *gallon*λ (DE3 [*lacIlac*UV5-T7 gene *1ind1sam7nin5*])	[9]
DH5α (pRK2013)	Helper strain used for the triparental mating; Km^R	[10]
Agrobacterium tumefaciens		
GV3101	Wild type; Rif^R	[11]
Salmonella enterica **serovar** ***thiphymurium***		
14028	Wild type strain	[12]
MPO96	14028Δ*trg*:: (*nahR*/Psal–*xylS2–nasR*/Km); Km^R	[13]
MPO386	14028Δ*sifA* Δ*trg*:: (*nahR*/Psal–*xylS2–nasR*/Km); Km^R	[14]
**Plasmids**		
pGEX-4T-1	GST fusion vector; Ap^R	GE Healthcare
pDONR207	Gateway donor vector; Gm^R	Invitrogen
pEarleyGate 100	Gateway-compatible plant transformation vector; Km^R	[15]
pEarleyGate 101	Gateway-compatible plant transformation vector with YFP and HA C-terminal tags; Km^R	[15]
pEarleyGate 104	Gateway-compatible plant transformation vector with a YFP N-terminal tag; Km^R	[15]
pMUS1135	pGEX-4T-1 plasmid carrying the HH103 *nopL* ORF cloned into the *Bam*HI/*Eco*RI restriction sites; Ap^R	This study
pMUS1243	pDONR207 carrying the HH103 *nopL* ORF without the stop codon; Gm^R	This study
pMUS1250	pEarleyGate 101 carrying the HH103 *nopL* ORF fused to YFP and HA; Km^R	This study
pMPO1003	Expression vector with rrnBT1T2-Pm-T7 SD sequence-MCSII-HA epitope encoding sequence, ColE1 replication origin; Ap^R	[13]
pMPO1004	Expression vector with rrnBT1T2-Pm-T7 SD sequence-MCSII-sspH2 signal peptide-HA epitope encoding sequence, ColE1 replication origin; Ap^R	[13]

pMPO1617	Expression vector with rrnBT1T2-Pm-T7 SD sequence-MCSII-NopL-HA epitope encoding sequence, ColE1 replication origin; Ap^R	This study
pMPO1631	pWSK29 derived plasmid with Pbla-tetR (no lysis plasmid); Cm^R	[14]
pMPO1632	pWSK29 derived plasmid with Pm-SRRz and Pbla-tetR (lysis plasmid); Cm^R	[14]
pcDNA5/FRT/TO-Venus-Flag (865)	pcDNA5/FRT/TO backbone with Venus YFP-3xFlag inserted via *Bst*XI-*Xho*I sites; Ap^R	Addgene
pMPO1643	pcDNA5/FRT/TO with fusion Venus-NopL; Ap^R	This study

* Ap^R, Nx^R, Gm^R, Km^R and Rif^R indicate resistance to ampicillin, nalidixic acid, gentamicin, kanamycin and rifampicin, respectively.

Supplementary Table S2. DNA oligonucleotide primers used in this study.

**Name**	**Sequence[1] 5′-3′**	**Use**
nopLEX_F	TTAGGATCCGATATCAATTCAACCCGC	Used for cloning of the *nopL* ORF in plasmid pGEX-4T-1 into *Bam*HI and *Eco*RI restriction sites
nopLEX_R	AACGAATTCTCAAATGTCAAAATCCAG	
pDONR_F	CGTTAACGCTAGCATGGATCTC	Sequence verification of inserts cloned into pDONR207
pDONR_R	GTAACATCAGAGATTTTGAGAC	
attB1_nopL	GGGGACAAGTTTGTACAAAAAAGCAGGCTTAATGGATATCAATTCAACC	Used for cloning of the *nopL* ORF in plasmid pDONR207 by the Gateway system
attB2_nopL	GGGGACCACTTTGTACAAGAAAGCTGGGTAAATGTCAAAATCCAGCGA	
NopL Fw/NdeI	AGCATATGGATATCAATTCAACC	Used for cloning of the *nopL* ORF in plasmid pMPO1003 into *Nde*I and *Sal*I restriction sites
NopL Rev/SalI	TAGTCGACAATGTCAAAATCCAGC	
NopL Fw/BamHI	CTGGATCCATGGATATCAATTCAACC	Used for cloning of the *nopL* ORF in plasmid pcDNA5/FRT/TO-Venus-Flag (865) into *BamH*I and *Not*I restriction sites
NopL Rev/NotI	TCGCGGCCGCTCAAATGTCAAAATC	

Underlined nucleotides indicate the restriction sites of the corresponding enzymes in the primer names or the attB1 or attB2 sequences indicated in the primer names.

References

1. Chung, C., Niemela, S.L., Miller, R.H. One-step preparation of competent *Escherichia coli*: transformation and storage of bacterial cells in same solution. *Proc. Natl. Acad. Sci.* **1989**, *6*, 2172-2175.
2. Sambrook, J., Russell, D.W. *Molecular cloning. A laboratory manual.* Cold Spring Harbor Laboratory Press: Cold Spring Harbor, USA, 2001.
3. Smith, D.B.; Johnson, K.S. Single-step purification of polypeptides expressed in Escherichia coli as fusions with glutathione S-transferase. *Gene*. **1988**, *15*, 31-40.
4. Bradford, M.M. A rapid and sensitive method for the quantitation of microgram quantities of protein utilizing the principle of protein-dye binding. *Anal. Biochem.* **1976**, *7*, 248-54.
5. Laemmli, U.K. Cleavage of structural proteins during the assembly of the head of bacteriophage T4. *Nature* **1970**, *15*, 680-685.
6. Monreal, J.A.; Arias-Baldrich, C.; Pérez-Montaño, F.; Gandullo, J.; Echevarría, C.; García-Mauriño, S. Factors involved in the rise of phosphoenolpyruvate carboxylase-kinase activity caused by salinity in sorghum leaves. *Planta*. **2013**, *237*, 1401-1413.
7. Jiménez-Guerrero, I.; Pérez-Montaño, F.; Monreal, J.A.; Preston. G.M.; Fones, H; Vioque, B.; Ollero, F.J.; López-Baena, F.J The Sinorhizobium (Ensifer) fredii HH103 Type 3 secretion system suppresses early defense responses to effectively nodulate soybean. *Mol. Plant. Microbe. Interact*. **2015**, *28*, 790–799.
8. Madinabeitia, N.; Bellogín, R.A.; Buendía-Clavería, A.M.; Camacho, M.; Cubo, T.; Espuny, M.R.; Gil-Serrano, A.M.; Lyra, M.C.; Moussaid, A.; Ollero, F.J.; et al. Sinorhizobium fredii HH103 has a truncated nolO gene due to a -1 frameshift mutation that is conserved among other geographically distant S. fredii strains. *Mol. Plant. Microbe Interact*. **2002**, *15*, 150-159.
9. Studier, F.W.; Moffatt, B.A. Use of bacteriophage T7 RNA polymerase to direct selective high-level expression of cloned genes. *J. Mol. Biol.* **1986**, *5*, 113-130.
10. Figurski, D.H.; Helinski, D.R. Replication of an origin-containing derivative of plasmid RK2 dependent on a plasmid function provided in trans. *Proc. Natl. Acad. Sci.* **1979**, *76*, 1648-1652.
11. Rotino, G.L.; Gleddie, S. Transformation of eggplant (Solanum melongena L.) using a binary Agrobacterium tumefaciens vector. *Plant Cell Rep.* **1990**, *9*, 26-29.
12. Fields, P. I.; Swanson, R. V.; Haidaris, C. G.; Heffron, F. Mutants of Salmonella typhimurium that cannot survive within the macrophage are avirulent. *Proc. Natl. Acad. Sci.* **1986**, *83*, 5189-5193.
13. Medina, C.; Camacho, E.M.; Flores, A.; Mesa-Pereira, B.; Santero, E. Improved Expression Systems for Regulated Expression in Salmonella Infecting Eukaryotic Cells. *PLoS One* **2011**, *6*, e23055.
14. Camacho, E.M.; Mesa-Pereira, B.; Medina, C.; Flores, A.; Santero, E. Engineering Salmonella as intracellular factory for effective killing of tumour cells. *Sci. Rep.* **2016,** *6*, 30591.
15. Earley, K.W.; Haag, J.R.; Pontes, O.; Opper, K.; Juehne, T.; Song, K.; Pikaard, C.S. Gateway-compatible vectors for plant functional genomics and proteomics. *Plant J.* **2006**, *45*, 616-629.
